# Hepatitis E Epidemic, Uganda

**DOI:** 10.3201/eid1601.090764

**Published:** 2010-01

**Authors:** Eyasu H. Teshale, Christopher M. Howard, Scott P. Grytdal, Thomas R. Handzel, Vaughn Barry, Saleem Kamili, Jan Drobeniuc, Samuel Okware, Robert Downing, Jordan W. Tappero, Barnabas Bakamutumaho, Chong-Gee Teo, John W. Ward, Scott D. Holmberg, Dale J. Hu

**Affiliations:** Centers for Disease Control and Prevention, Atlanta, Georgia, USA (E.H. Teshale, C.M. Howard, S.P. Grytdal, T.R. Handzel, V. Barry, S. Kamili, J. Drobeniuc, C.-G. Teo, J.W. Ward, S.D. Holmberg, D.J. Hu); Ministry of Health, Republic of Uganda, Kampala, Uganda (S. Okware); Global AIDS Program, Kampala (R. Downing, J.W. Tappero); Ugandan Virus Research Institute, Entebbe, Uganda (B. Bakamutumaho)

**Keywords:** Viruses, hepatitis virus, epidemic, Uganda, dispatch

## Abstract

In October 2007, an epidemic of hepatitis E was suspected in Kitgum District of northern Uganda where no previous epidemics had been documented. This outbreak has progressed to become one of the largest hepatitis E outbreaks in the world. By June 2009, the epidemic had caused illness in >10,196 persons and 160 deaths.

Hepatitis E virus (HEV) infection causes large epidemics of liver disease in developing countries (*1*–*3*). In epidemic settings, HEV is transmitted bythe fecal–oral route, and the most commonly attributed source of infection is feces-contaminated drinking water (*4*). The incubation period after exposure ranges from 3 to 8 weeks (mean 40 days) and is dose dependent (*5*,*6*). Illness is generally self-limited, with death rates <4% in the general population (*7*), but a strikingly high death rate (10%–25%) has been reported among pregnant women (*8*).

In October 2007, an epidemic of hepatitis E was suspected in northern Uganda, where no previous epidemics had been documented. However, outbreaks of hepatitis E had occurred in neighboring Sudan and Chad in 2004 (*9*,*10*). Beginning in the Madi Opei subcounty of Kitgum District, this outbreak has progressed to become one of the largest hepatitis E outbreaks in Africa and globally. By June 2009, a year after the study we report here, the epidemic involved all 19 subcounties of Kitgum and had caused illness in >10,196 persons and 160 deaths (local surveillance, unpub. data). This report describes the results of a case finding and seroprevalence survey in 2 subcounties of Kitgum District, Madi Opei and Paloga.

## The Study

Two subcounties, Madi Opei and Paloga, were selected for a census and seroprevalence survey. In June 2008 (at the time of this investigation), the 2 subcounties represented different stages of the epidemic: Madi Opei was the first subcounty to experience the epidemic; the epidemic appeared to affect Paloga relatively later. No differences in demographic and socioeconomic characteristics were evident between the residents of the 2 subcounties. Trained village health team members conducted a hut-to-hut census. The census was completed using a standardized data collection instrument. Histories of jaundice and jaundice-related death were also obtained.

For the seroprevalence survey, a random sample of residents was identified from the list created during the census. Persons who consented to participate had blood collected by venipuncture. Blood specimens were tested for immunoglobulin (Ig) M and IgG against HEV (MP Biomedicals Asia Pacific Pte Ltd, Singapore), HEV RNA (by an in-house reverse transcription–PCR assay), and serologic markers of infection by hepatitis A virus, hepatitis B virus, and hepatitis C virus. A subset was analyzed to determine the HEV genotype. The HEV sequence from this outbreak strain was compared with other HEV genotype 1 strains isolated from past epidemics. Because the 2 sites were in different stages of the outbreak as determined by the different durations and peaks of the epidemic at the time of the investigation (June 2008), HEV attack and death rates were calculated separately by site and in aggregate. All statistical analyses were performed with SAS version 9.1 (SAS Institute, Inc., Cary, NC, USA).

**Figure 1:**
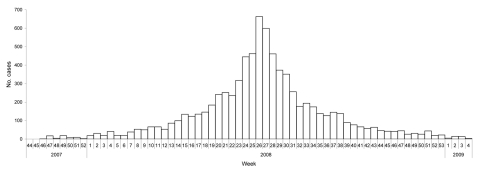
Distribution of cases of jaundice during an epidemic of hepatitis E in Kitgum District, Uganda (N = 7,919), by week of report, October 2007 through January 2009.

**Figure 2:**
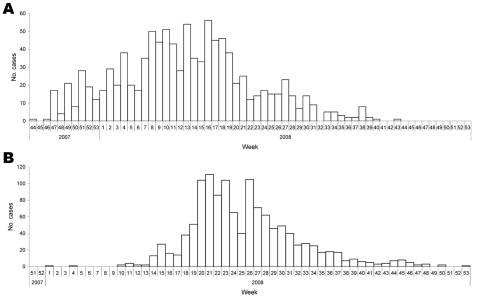
Distribution of cases of jaundice during an epidemic of hepatitis E in A) Madi Opei subcounty (n = 1,026) and B) Paloga subcounty (n = 1,248), by week of report, Kitgum District, Uganda, October 2007 through January 2009. Data are from facility-based passive surveillance.

A total of 19,098 persons were counted in Madi Opei (10,535) and Paloga (8,563 during the census. In Madi Opei, there were 2,137 families and an average of 4.9 persons per household. In Paloga, there were 1,884 families and an average of 4.5 persons per household. Figure 1 shows the distribution of cases of jaundice in Kitgum District, by week of report, October 2007 through January 2009 (data from facility-based passive surveillance). The overall symptomatic hepatitis E attack rate, based on hut-to-hut case finding, in the 2 subcounties was 25.1%. However, at the time of the investigation, the epidemic had peaked (Figure 2, panel A) in Madi Opei and was still increasing in Paloga: 30.1% of Madi Opei residents reported jaundice, but only 18.9% of Paloga residents reported jaundice by the time of the investigation. Symptomatic cases reached their height in April 2008 in Madi Opei but did not peak in Paloga until June 2008 (Figure 2, panel B).

Of the 10,535 Madi Opei residents, jaundice was reported by 3,170 (30.1%). In Paloga, jaundice was reported by 1,619 (18.9%) of 8,563 residents. The number of symptomatic cases was higher for women (28%) than for men (22%; p<0.001) (Table). The symptomatic attack rate was lowest for children <2 years of age (6.9%) and highest for pregnant women (80.7%). In the 2 subcounties, 72 deaths were reported among 4,789 persons with jaundice, yielding an estimated case-fatality rate among jaundice cases of 1.5%. Among the 72 jaundice-related deaths, a disproportionate number occurred in children <2 years of age (12/92, 13%) and in pregnant women (13/189, 6.9%).

**Table:** Symptomatic and serologic evidence of HEV infection, Uganda, June 2008

Characteristic	Jaundice			HEV seropositivity	
	No. examined	No. (%) with jaundice		No. tested	No. (%) seropositive
Age group, y					
<2	1,352	92 (6.8)		23	7 (30.4)
2–4	2,213	370 (16.7)		49	28 (57.1)
5–9	3,361	589 (17.5)		59	36 (61.0)
10–14	2,692	504 (18.7)		70	49 (70.0)
15–44	7,155	2,402 (33.6)		189	135 (71.4)
≥45	2,186	816 (37.3)		75	48 (64.0)
Sex					
M	9,177	2,017 (22.0)		188	122 (64.9)
F	9,877	2769 (28.0)		277	180 (65.0)
Total	19,098	4789 (25.1)		469	305 (65.0)

*HEV, hepatitis E virus.

Sixty-six percent of 720 randomly selected residents agreed to participate in the survey and blood draw. Of the total tested, 305 (64.4%) were positive for IgM or IgG against HEV or both. In a subset of 142 specimens selected at random from among the participants of the survey and tested by reverse transcription–PCR, 24 were found to be positive for HEV RNA. Subsequent sequence analysis showed that HEV found in all 24 specimens belonged to genotype 1. There was close resemblance of the open reading frame 2 gene of the HEV genotype 1 isolates from this and the Chad outbreak. Other causes of viral hepatitis were rare. Of 469 persons tested, only 12 (2.5%) tested positive for IgM to hepatitis A virus, 3 (0.6%) were positive for IgM to hepatitis B core antigen, and 4 (0.8%) were positive for antibody to hepatitis C virus.

## Conclusions

The symptomatic HEV attack rate and HEV-related deaths observed in this epidemic were high. Given that outbreaks of hepatitis E had not been observed or reported in Uganda previously, a lack of preexisting immunity from prior exposure in this population may well have facilitated the outbreak. Nevertheless, outbreaks had been reported in neighboring countries and, although there were no sequences available from the epidemic in southern Sudan, phylogenetic-relatedness analysis showed close resemblance of the outbreak strain to the strain from the Chad outbreak. There was no clear epidemiologic link of this outbreak to the neighboring countries.

Women in this epidemic were substantially affected, and, as seen in previous epidemics, pregnant women are particularly at increased risk of death (*4*). Furthermore, our survey also showed that children (0–2 years of age) were at a higher risk of dying from hepatitis E, despite the fact they were generally asymptomatic. This finding corroborates a similar observation for a hepatitis E epidemic in the former Soviet Union in 1985–1987 (*11*). We do not know why pregnant women and young children were at increased risk for death from this infection, but there may be gender- or age-specific risk for exposure or differential susceptibility to infection (*1*,*7*).

This investigation has some limitations. For the census, the clinical diagnosis of hepatitis E was by self-report only, and the death rate data were based on verbal autopsy. Jaundice could have been overdiagnosed for young children and thus could have contributed to a skewed death rate being reported for this group.

Current understanding of HEV transmission indicates that effective prevention and control depend on ensuring a safe drinking water supply, adequate sanitation, and proper personal and environmental hygiene. However, due to the rapid transmission of HEV and the long incubation period of this disease, it is difficult to mount adequate prevention measures in a timely manner. This difficulty was evident in the long duration of the outbreak in Kitgum district. Therefore, we recommend that increased priority be given to developing a promising current hepatitis E vaccine candidate as soon as possible (*12*). Availability of vaccine is also needed in light of the high death rate of children and pregnant women. However, the safety of such a vaccine for pregnant women needs to be determined before use in this population.
